# A Review on AC-Dielectrophoresis of Nanoparticles

**DOI:** 10.3390/mi16040453

**Published:** 2025-04-11

**Authors:** Tonoy K. Mondal, Aaditya V. B. Bangaru, Stuart J. Williams

**Affiliations:** Department of Mechanical Engineering, University of Louisville, Louisville, KY 40208, USA; t0mond03@louisville.edu (T.K.M.); aadityavb001@gmail.com (A.V.B.B.)

**Keywords:** nanoparticles, dielectrophoresis, nanomanipulation, microfluidics, microelectrodes, nanoelectrodes, colloids

## Abstract

Dielectrophoresis at the nanoscale has gained significant attention in recent years as a low-cost, rapid, efficient, and label-free technique. This method holds great promise for various interdisciplinary applications related to micro- and nanoscience, including biosensors, microfluidics, and nanomachines. The innovation and development of such devices and platforms could promote wider applications in the field of nanotechnology. This review aims to provide an overview of recent developments and applications of nanoparticle dielectrophoresis, where at least one dimension of the geometry or the particles being manipulated is equal to or less than 100 nm. By offering a theoretical foundation to understand the processes and challenges that occur at the nanoscale—such as the need for high field gradients—this article presents a comprehensive overview of the advancements and applications of nanoparticle dielectrophoresis platforms over the past 15 years. This period has been characterized by significant progress, as well as persistent challenges in the manipulation and separation of nanoscale objects. As a foundation for future research, this review will help researchers explore new avenues and potential applications across various fields.

## 1. Introduction

Numerous cutting-edge material and device innovations, including tunable materials, reconfigurable devices, photonic materials, and meta-materials, may be made possible by the capacity to control nanoscale particulates [1,2,3]. A wide range of nano particulates, including nanoparticles (NPs) [4,5], nanowires (NWs) [6], graphene [7], and others [8,9,10,11], are the fundamental building blocks utilized to produce nanoscale goods. There are several approaches to manipulate NPs including using electric fields [12], magnetic fields [13], hydrodynamic fields [14], and the fields produced by chemically active substances [15]. Among the most widely used methods are optical and magnetic tweezers, which are based on their respective field gradients [16].

Hydrodynamic fields use only hydrodynamics for particle manipulation. For example, deterministic lateral displacement uses tilted pillar arrays to create unique flow streamlines based on the particle size. Although easy to implement, the numerous pillars can cause low throughput and channel clogging. For NPs, hydrodynamic field efficiency is further diminished by diffusion effects [17]. Being non-contact, having a high throughput isolation, being low-cost, having selectable controllability, and having low heat generation are all benefits of magnetic fields [18]. Despite of all these advantages, manipulating NPs using magnetic fields is limited in applications because it necessitates the employment of magnetic particles. Furthermore, the load capacity of the magnetic beads determines the separation efficiency, and sample preparation is labor-intensive and time-consuming [19]. Additionally, the integrity of biomolecules may be impacted by the buildup of magnetic nanoparticles and extended exposure to the paramagnetic medium [18]. A delicate balance in the optical contrast between the medium and the particles is necessary for the assembly of optical fields, making their use restrictive; furthermore, adapting nanoscale optical systems for high throughput nanoparticle manipulation is not trivial because the optical force is generally weaker than other forces [20,21]. Additionally, because of concerns about biocompatibility, the application to biological particles is limited by excessive heat generation during optical illumination [22]. To learn more about different techniques of nanoparticle separation, readers are referred to this review article [23].

**Figure 1:**
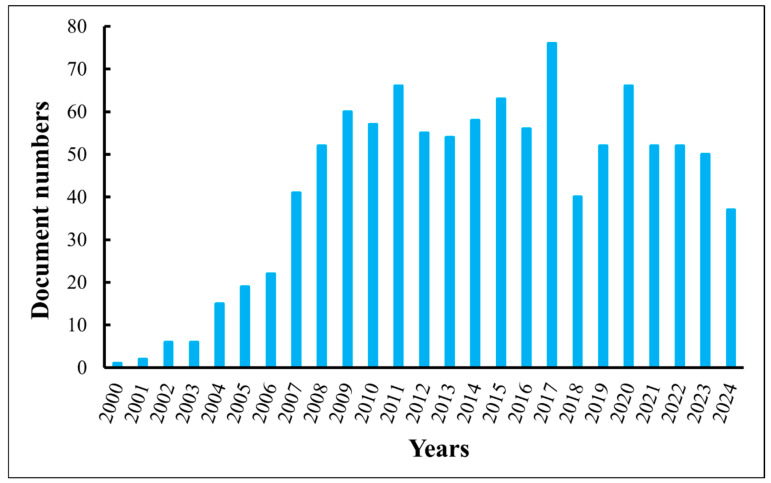
The number of papers published in nanoparticles DEP field over time. The data came from an open-source, date-restricted Web of Science search for “dielectrophoresis” and “nanoparticles”. After 2010, the number of yearly publications has remained largely unchanged.

Dielectrophoresis (DEP)-based nanoscale material manipulation [24,25,26], on the other hand, has attracted a lot of attention since the beginning of 21st century, and the number of published documents has remained significantly unchanged from 2010 to the present, as shown in Figure 1.

DEP has several advantages for the manipulation of nanomaterials because of it being label-free, non-invasive, and inexpensive [27]. Nevertheless, there are still certain difficulties in producing a sufficient non-uniform electric field at the nanoscale and manipulating nanomaterials on a large, flexible, and accurate scale. It is encouraging that researchers from a wide range of disciplines have worked towards increasing the precision, adaptability, and scale of DEP manipulation of nanomaterials. This has created a solid basis for studies into precise nano-surgery, drug delivery, nanoscale manufacturing systems, and other topics [23,28,29,30,31,32]. There are several outstanding review articles on this topic regarding the theory, development, and applications of DEP-based systems [27,32,33,34,35,36,37]. However, they have either not emphasized the differences between nanoparticle and microparticle manipulation using DEP or have not described the current state-of-the-art applications.

DEP manipulates particles through the interaction of an induced dipole and a non-uniform electric field. The creation of metal and/or insulator structures that can produce electric field gradients strong enough to manipulate and trap NPs became conceivable due to rapid advancements in micro- and nanofabrication techniques. This review will include studies published from 2010 to date where researchers extensively focused on AC-DEP systems where at least one dimensions of the geometry and/or manipulated particles are equal to or less than 100 nm. The fundamentals of AC-DEP at this scale are discussed in Section 2. Section 3 covers an extensive overview of nanoparticle-based DEP system development through novel applications including assembly, manipulation, separation, and trapping. In addition, Section 3.1,Section 3.2,Section 3.3,Section 3.4 categorizes studies based on nanoparticle type including metallic, non-conductive, NWs and similar, and others. Finally, in Section 4, we conclude by examining the remaining obstacles and prospects for the further development of DEP-based systems for NPs.

## 2. Theory of AC-DEP

In DEP a non-uniform electric field applies a force to a polarizable particle. This ponderomotive force was investigated by Boltzmann [38] and further investigated experimentally by Pohl in 1951, with the latter terming this phenomenon ”dielectrophoresis”. The electric field induces surface charges in the polarizable object, which form a dipole (or higher ordered poles). The frequency and strength of the applied electric field, the shape of the particles, and the dielectric characteristics of the medium and particles all affect the particles’ polarizability [24]. In a uniform electric field, the net force resulting from Coulomb interactions is zero. However, in a non-uniform electric field, the net force acting on a particle is non-zero. This force depends on the spatial non-uniformity of the field itself and its magnitude. DEP is divided into two cases: positive and negative [39]. A particle in positive DEP is driven towards an area of a higher electric field because it is more polarizable than the surrounding medium. A phenomenon known as negative DEP occurs when a suspended particle is pulled towards an area with a weaker electric field if its polarizability is less than that of the surrounding medium. Either direct current (DC) or alternating current (AC) electric fields can cause DEP. The induced force’s direction remains constant in an AC electric field at a constant field frequency.

The time averaged DEP force acting on a homogeneous sphere can be described by the following expression [24,25,26]:

(1) $$F_{DEP}=2\pi \varepsilon_{m}a^{3}Re(CM)\nabla {\left|E\right|}^{2}$$

The magnitude of the DEP force exerted on a particle depends proportionally on the following characteristics: (1) medium permittivity ($\varepsilon_{m}$), (2) particle volume ($a^{3}$, where *a* is particle radius), (3) real part of the Clausius–Mossotti factor (Re(CM)), (4) the gradient of the electric field-squared ($\nabla {\left|E\right|}^{2})$, where *E* is the rms of the applied electric field. For the case of very small particles (NPs), the particle volume component of Equation (1) is weakened; to cope up with inherent smaller DEP forces on NPs, researchers often utilize smaller and sharp electrode features to generate significant electric field gradients [33].

The frequency-dependent Clausius–Mossotti factor represents the value of the induced dipole moment due to the interaction between the NPs and the medium, and can be represented as follows [40]:

(2) $$CM\left(\omega \right)=\frac{{\tilde{\varepsilon }}_{p}-{\tilde{\varepsilon }}_{m}}{{\tilde{\varepsilon }}_{p}+2{\tilde{\varepsilon }}_{m}}$$

(3) $$\tilde{\varepsilon }=\varepsilon -j\frac{\sigma }{\omega }$$

where $\tilde{\varepsilon }$ is the complex permittivity and subscripts *p* and *m* refer to the particle and medium, respectively, angular frequency *ω* is 2π*f*, electrical conductivity and permittivity are *σ* and ε, respectively, and *j* is $\sqrt{-1}$. Re(CM) has a theoretical range of −0.5 to +1.0, where the sign dictates whether the particle experiences positive or negative DEP.

For small particles, the particle conductivity can be expressed as follows:

(4) $$\sigma_{p}=\sigma_{b}+\frac{2K_{s}}{a}$$

where *σ_b_* is the bulk conductivity of the particles, $\frac{2K_{s}}{a}$ is the surface conductivity brought on by the charges in the Stern layer and the diffused double layer [41], and $K_{s}$ is the surface conductance. These induced shell-like layers can affect the DEP behavior because they differ from the bulk particles’ dielectric characteristics [42]. In particular, this impacts low and/or non-conductive materials (like polystyrene) such that the surface conductivity becomes crucial [43]. Therefore, changing fluid conductivity not only changes $\sigma_{m}$ but also changes $\sigma_{p}$ as well, which alters the frequency-dependent Clausius–Mossotti factor.

According to Equation (2), positive DEP (pDEP) occurs when the particle is more polarizable than the medium (${\tilde{\varepsilon }}_{p}>{\tilde{\varepsilon }}_{m}$) and particles translate to stronger fields. Instead, if ${\tilde{\varepsilon }}_{p}<{\tilde{\varepsilon }}_{m}$, the particle is repelled from strong field regions, which is called negative DEP (nDEP). When ${\tilde{\varepsilon }}_{p}={\tilde{\varepsilon }}_{m}$, $Re\left(CM\right)=0$ and the particles will not experience DEP force and it occurs at a crossover frequency (*f_cv_*) [44], which is expressed by the following:

(5) $$f_{CV}=\frac{1}{2\pi }\sqrt{\frac{\left(\sigma_{m}-\sigma_{p}\right)\left(\sigma_{p}+{2\sigma }_{m}\right)}{\left(\varepsilon_{p}-\varepsilon_{m}\right)\left(\varepsilon_{p}+{2\varepsilon }_{m}\right)}}$$

Many DEP particle separation schemes take advantage of the frequency-dependent nature of $Re\left(CM\right)$; for example, sorting different dielectric particles where one exhibits pDEP and the other nDEP at a specific frequency. Particles of similar dielectric properties can be separated based on size since the DEP force is proportional to particle volume.

Since the *gradient* of the field-squared determines the DEP force’s magnitude and direction, it is possible to use both DC and AC fields. DC can simultaneously induce electrophoretic affects and their combined effects can be utilized [45]. AC fields reduce electrophoretic effects, particularly at moderate frequencies (>1 kHz). Higher frequencies also reduce electrochemical reactions between the electrode and medium.

One of the most significant challenges associated with trapping NPs is overcoming their random thermal (Brownian) motion, which must be addressed in order to produce deterministic movement among distinct particles. An approximate representation of the influence of thermal motion is provided by a random force acting on a particle, referring the RMS Brownian displacement of a particle in one second as its effective Brownian motion velocity [24], which is calculated as follows:

(6) $$v_{B}=\sqrt{\frac{k_{B}T}{3\pi \eta a}}$$

where temperature is *T*, $k_{B}$ is Boltzmann’s constant, and *η* is fluid viscosity. To estimate if DEP forces could overcome Brownian motion, $v_{B}$ can be compared to a particle’s induced dielectrophoretic velocity, derived from Stokes’ drag for a sphere using the following equation:

(7) vDEP=FDEP6πηa

The required gradient of the field-squared for successful DEP trapping (i.e., $v_{DEP}\geq v_{B}$) for particles of different sizes suspended in water ($\varepsilon_{m}=80\varepsilon_{o}$, where $\varepsilon_{o}$ is the permittivity of free space) and when the $Re\left(CM\right)=1.0$ is shown in Table 1. The need for significant values for the gradient of the field-squared ($\nabla {\left|E\right|}^{2}$) for NPs is apparent. Increasing the applied voltage is one approach, as *F_DEP_* is proportional to voltage-squared ($V^{2}$); however, larger voltages have a risk of triggering electrolysis and significantly increasing Joule heating (which is proportional to $\sigma_{m}V^{2}$) [26]. Therefore, researchers in the area of nanoparticle DEP trapping have developed innovative techniques to enhance their system’s $\nabla {\left|E\right|}^{2}$ (see Section 3).

The device’s hydrostatic pressure often produces the required fluid flow, generating a laminar Poiseuille flow [46] whose parabolic flow profile has maximum velocity in the center of the channel and a no-slip condition at its edges. Fluid can also be driven by an electric field in addition to a hydrostatic pressure differential. The application of an electric field induces electrohydrodynamic mechanisms as a result of the solvent’s mobility in an electric field [47]. One important electrohydrodynamic phenomenon is the electrothermal (ET) flow that results from dynamic changes in net charge density, which leads to variations in the fluid’s permittivity and electrical conductivity. Spatial property variations are caused by non-uniform temperature fields that are generated by Joule heating or external heating sources (ex: illumination). A temperature rise results in a decrease in permittivity and an increase in local conductivity. To maintain charge conservation, it is necessary to lower the local electric field. However, reducing the local electric field also requires the presence of a local charge density, in accordance with Gauss’s law [48]. Consequently, variations in net charge density create an electrostatic body force. The time average ET fluid body force is given by [49], and is as follows:

(8) $$F_{e}=\frac{1}{2}\varepsilon_{m}\left[\left.\left(C_{\varepsilon }-C_{\sigma }\right)\frac{\left(\nabla T\cdot E\right)}{1+{\left(\omega \tau \right)}^{2}}E-\frac{1}{2}C_{\varepsilon }\nabla T{\left|E\right|}^{2}\right]\right.$$

where $\tau =\frac{\varepsilon_{m}}{\sigma_{m}}$ is the charge relaxation time of the fluid. Constants $C_{\varepsilon }$ and $C_{\sigma }$ are the linear approximations of the temperature dependence of the electrical permittivity and conductivity, respectively. The flow magnitude is highly reliant on media conductivity and is typically considerable for electrolyte conductivities more than 100 mS/m [50]. Higher voltages result in stronger flows at sufficiently large conductivities because of a stronger temperature gradient, as electrothermal flow caused by Joule heating is proportional to $\sigma_{m}V^{4}$. There are two distinct limiting scenarios for the force density, which is dependent on the AC frequency. Equation (8)’s left term, the Coulomb force, dominates at low frequencies (*ωτ* ≪ 1), whereas the second term, the dielectric force, dominates at high frequencies. Depending on the charge relaxation time of the fluid, these two forces usually act in separate directions, affecting the flow pattern [51]. However, in many cases, the high-frequency regime of ET flow is weak or negligible [52,53].

AC electro-osmotic (ACEO) flow is another type of fluid flow caused by an electric field. The Coulomb force is the source of electro-osmosis, which is the result of an electric field interacting with free charges in the electric double layer on the surface of the electrode [54]. The applied electric field causes charges in the diffuse layer to move in the tangential direction of the field, creating a drag flow in the fluid. This results in induced fluid motion along the surface of the electrode. This phenomenon can also occur on metallic or conductive surfaces that are ‘floating’ and are not the electrodes themselves, called induced charge electro-osmosis (ICEO) [55]. In general, ACEO and ICEO are significant at low frequencies (<10 kHz) and in low-conductivity media (<1 mS/m), and they may be negligible for high frequencies and/or moderate fluid conductivities. Both ACEO and ET flow can be used constructively to enhance DEP trapping [56].

For certain applications, such as bio-sample processing, managing or reducing thermal effects is crucial to enhance the viability and biocompatibility. Joule heating, which refers to the heating of the medium and the system due to an electric current, is proportional to $\sigma_{m}V^{2}$. Consequently, applying a high-voltage electric field in a conductive medium can lead to increased temperatures within the system. To mitigate temperature-related stress, the conductivity of the medium or buffer must be designed to minimize temperature rise [57]. Although using a lower voltage can help reduce Joule heating, it may also decrease the efficacy of DEP by lowering the electric field gradient. To address this challenge, it is important to minimize the overall dimensions of the electrodes, including their spacing. Additionally, constructing electrodes with small, sharp features can concentrate the electric field, thereby increasing the field gradient even when using lower voltages. Implementing insulation on the electrodes, such as coatings, can act as a barrier between the electrodes and the fluid [58]. Using materials with high thermal conductivity in chip design can also help to dissipate the heat generated by electric field-driven heating more effectively [53,59]. The time-averaged DEP force mentioned in Equation (1) is applicable for homogeneous spherical particles with a thin double layer. When dealing with thick double layers, the Poisson–Nernst–Planck equation must be solved as it describes ion transport resulting from both concentration gradients and electric fields. This is important because, at moderate to high double-layer thicknesses, polarization is influenced by the electrophoretic motion of the particles. Zhao et al. provided a comprehensive review of this topic [60]. In the case of non-homogeneous particles, particularly complex bioparticles, researchers often utilize a shell model that accounts for the different dielectric properties of various shells, which can result in multiple crossover frequencies [26]. For charged particles, it is also essential to consider counter-ion polarization, which occurs between the charged particles and their respective counter-ions from the solvent [61]. This effect is only significant when the time scale is much shorter than the relaxation time of the counter-ions. For example, the polarization of the electrical double layer and the interactions between solvents and proteins contribute to the overall polarization and dipole moment of proteins [62].

## 3. DEP of Nanoparticles

For the manipulation, assembly, sorting, and trapping of NPs, one needs to achieve a significant field gradient compared to that for micron-sized particles (Table 1). As the distance between electrodes increases, the electric field gradient exponentially decreases [63]. Therefore, the gap between the electrodes must be reduced and optimized to induce electric field gradients sufficient to manipulate NPs with DEP. Advances in microfabrication techniques enable the creation of precise and diverse materials at micro/nanoscale geometries to produce on-demand electric field gradients [64]. Even though nanoscale features may require advanced techniques like electron beam lithography, shrinking electrode features have several benefits. First, miniaturization reduces the spacing between electrodes, significantly lowering Joule heating and the occurrence of electrolysis and/or electrochemical degradation due to a lower applied voltage to achieve similar field strengths. Second, as the DEP force depends on the gradient of the square of the electric field, the DEP force acting on the nanoscale geometries increases significantly at these smaller scales, and increases considerably more for 3D electrodes [65].

For electrode-based AC-DEP, which is the most traditional application of DEP, electrodes are inside of the microchannel and are in direct contact with the particles and solution. Noble metals like platinum and gold remain at the forefront of fabricating electrodes because of their electrochemical stability. Indium tin oxide (ITO), fluorine-doped tin oxide (FTO), and other transparent conductors are used to help researchers visualize their experiments. A variety of geometrical patterns of electrodes and microchannel features utilize a variety of fabrication methods. Micrometer patterning of electrodes can be achieved with traditional photolithography; however, achieving sub-micrometer features often requires a combination of techniques including optical lithography, e-beam lithography, nanoimprint lithography, focused ion-beam lithography, atomic layer epitaxy, scanning probe lithography, molecular self-assembly, vapor-phase depositions, etc. [66,67,68,69]. Nanoscale electrode edges and corners are the most prevalent sites for positive dielectrophoretic trapping [70]. Common electrode geometries include interdigitated microelectrodes [71], quadruple electrodes [72], parallel electrodes [73], and nano-electrode arrays [74]. Of these, interdigitated electrode arrays are one of the most common due to their simple geometry (the DEP field can be modeled in 2D [75]) and ability to be patterned to produce large arrays. Electrode gaps and/or features should be as small as possible to generate large field gradients sufficient to trap NPs, and there are several examples of nanoscale planar electrodes [76,77,78].

Besides planar electrodes, nanoparticle DEP trapping can be achieved using novel electrode materials and/or fabrication techniques. For example, Brody and colleagues [79] suggested that carbon nanotubes (CNTs) are an ideal option for DEP nanoelectrodes because of their small diameter—1 nm for single-walled carbon nanotubes and a few nanometers for multi-walled carbon nanotubes. Other groups have also used CNTs as electrodes for DEP trapping [80,81]. Yu et al. [82] used nano-gap electrodes, where exposed metal layers were separated by a thin insulating layer. Other researchers have used extruded 2D structures like conductive NWs [83,84], and probes/tips [56,85]. Recently, Williams and co-workers have developed carbon nanofiber (CNF)-mat electrodes with a large surface area (>mm^2^) by electrospinning for bulk nanoparticle trapping using DEP, and these might be useful for low-cost fabrication and a high throughput of smaller particles [86,87]. The smaller features (~250 nm) of CNF mat generated a significant gradient of the field-squared to trap particles as small as 20 nm.

Regardless of the chosen method of DEP capture, there have been many studies on trapping NPs. This section focuses on the types of particles manipulated with DEP, divided according to their properties: (1) metallic NPs which are made of electrically conductive materials like Au, Pt, etc.; (2) non-conducting NPs usually made of plastics or polymers like polystyrene, etc., including quantum dots (QDs); (3) NWs/NTs/NRs and similar one-dimensional materials; (4) other nanomaterials like semiconductive carbon black, graphene, etc. NPs exhibit special qualities, including mechanical (such as exceptional resilience), electrical (such as high carrier mobility), physical (such as a high surface-to-volume ratio), optical, and/or chemical features. In order to effectively trap or manipulate NPs in bulk using DEP, it is essential to carefully adjust and optimize parameters such as the electric field voltage, frequency, and the conductivity of the solution.

### 3.1. Metallic Nanostructures

Assembled NPs provide enhanced surface area, tunable optical properties, and have excellent electrical conductivity. Researchers have used them to create sensitive electrochemical sensors [88] or enhanced optical detection [89]. In particular, gold NPs (AuNPs) are extensively used in bio-applications due to their versatility and biocompatibility [90]. Therefore, several researchers have sought to utilize DEP as a tool to handle and/or assemble metallic NPs to enhance these applications. A summary of the relevant works is listed in Table 2.

**Figure 2:**
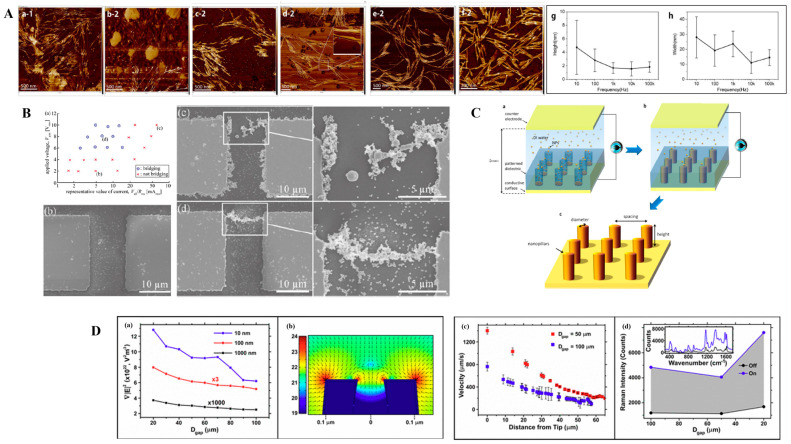
(**A**) Effect of frequency on nanoribbons development with applied frequency of (**a**-**1**) 100 Hz, (**b**-**2**) 1 kHz, (**c**-**2**) 10 kHz, (**d**-**2**) 100 KHz, (**e**-**2**) 1 MHz, (**f-2**) 10 MHz, (**g**) the distribution of height with frequency, and (**h**) the distribution of width with frequency [91]; (**B**) Au nanoparticle chain formation with 10 μm-wide gap: (**a**) formation condition plot between applied voltage (V_gap_) and representative value of current, SEM image in the case of (**b**) 2.0 V_rms_ with 4.9 mA_rms_, (**c**) 10.0 V_rms_ with 60 mA_rms_, and (**d**) 8.1 V_rms_ with 6.4 mA_rms_. Adapted and modified with permission from [93]. Copyright 2017 John Wiley and Sons. (**C**) Electric field-directed assembly of NPs towards fabricating 3D nanostructures: (**a**,**b**) NPs suspended in aqueous solution are (**a**) assembled and (**b**) fused in the pattern via geometries under an applied AC electric field, (**c**) removal of the patterned insulator film after the assembly. Adapted and modified with permission from [73]. Copyright 2014 American Chemical Society. (**D**) Influence of device configuration on trapping and SERS enhancement: (**a**) gradient of electric field-squared as a function of electrode separation, (**b**) gradient of field-squared logarithmic colormap, (**c**) particle tracking of AuNP at two different gaps, and (**d**) SERS measurement with and without DEP trapping. Adapted and modified with permission from [94]. Copyright 2016 American Chemical Society.

A significant advancement in this field is the development of nanoribbons from AuNPs on mica substrates through DEP-assisted cold welding (Figure 2A) [91]. This technique leverages the manipulation of electric field parameters, such as a voltage ranging from 1 to 20 V and frequencies between 100 Hz and 10 MHz, to control nanostructure growth. Adjusting the frequency led to the transformation of lengthy, winding ribbons into shorter, thinner, and straighter forms, highlighting the precision achievable with DEP. Additionally, platinum NPs assembled on reduced graphene oxide formed nanohybrids that greatly enhanced the performance of gas sensors, showcasing the potential of hybrid structures to achieve superior sensitivity [92].

DEP’s potential is further demonstrated in the precise positioning of single NPs. AuNPs have been trapped in nanogaps to fabricate single-electron transistors [95]. These devices rely on Coulomb blockade behavior, observed at low temperatures, to achieve high-precision electronic functionalities. The ability to position individual particles with such accuracy highlights DEP’s role in advancing nanoscale electronic applications.

Beyond assembly, DEP has revolutionized surface-enhanced Raman scattering by enabling the creation of hotspots, regions of intense localized electric fields that amplify Raman signals [96]. For example, researchers arranged gold NPs into pearl chains with nanogaps, using an AC voltage of 10 V_pp_ at 1 MHz, to detect molecules like adenine at femtomolar concentrations [71]. Silver NPs have also been dynamically positioned to create controlled hotspots such as dendrite, facilitating the detection of proteins and chemical biomarkers such as dipicolinate [97], melamine, and cocaine [4]. This active control of interparticle spacing underscores DEP’s versatility in tailoring structures for specific applications.

DEP has been instrumental in advancing the functionalization of scanning probe tools, particularly in enhancing Raman signals. Researchers have successfully positioned NPs at the apex of atomic force microscope (AFM) tips, enabling applications such as tip-enhanced Raman spectroscopy [74]. By fine-tuning DEP parameters, including the voltage and frequency, a reliable attachment of NPs has been achieved, resulting in high signal-to-noise ratios during Raman measurements [85]. The gap distance between electrodes can play a significant role for signal enhancement as well, as shown in Figure 2D [94]. This method not only improves detection accuracy but also offers a consistent approach for developing sophisticated sensing tools.

DEP’s ability to create one-dimensional nanoparticle chains has opened new frontiers in nanoelectronics and sensing. Chains of metallic NPs, formed through DEP, exhibit unique properties that are useful in flexible electronics, granular conductors, and bioelectronic applications [5]. For instance, researchers have shown that voltage and current conditions can be tuned to assemble chains of 150 nm AuNPs, as shown in Figure 2B, revealing dependencies on electrode gap width and electric field strength [93]. These findings provide insights into the design of conductive pathways and functional components for next-generation devices.

The controlled aggregation of metallic NPs extends to applications in biosensing and chemical detection. Concentrating NPs onto nanostructured tips using DEP has proven effective for detecting low-abundance analytes [98]. For instance, gold NPs as small as 80 nm were successfully trapped by leveraging both their polarizability and the transport effects of Brownian motion [56,99]. These advancements have significant implications for heat-sensitive applications, where low voltages and high frequencies ensure effective trapping without thermal degradation.

In more complex assemblies, DEP has enabled multi-step processes for constructing hetero-nanostructures. A two-step DEP process was used to fabricate Au-ZnO nanostructures for ultraviolet light detection, where the attachment of AuNPs significantly enhanced the photodetector’s performance [10]. Furthermore, 3D metallic nanostructures, such as nanopillars and nanorings, were assembled by optimizing DEP parameters, achieving electrical properties equivalent to electroplated gold and showcasing strong plasmonic resonances [73]. The schematic of the fabrication of 3D nanostructures is shown in Figure 2C.

**Table 2:** Summary of research works in DEP of metallic nanostructures.

Serial No.	Nanoparticle			Electric Field Signal		Electrode Information			Ref.
	NP Type	Size (Diameter/Length)	Material	Voltage	Frequency	Materials	Types	Length Scales (Gap/Length/Width/Height)	
1	Metal NPs	20 nm	Au	1–20 V	100 Hz–10 MHz	Al	Parallel, arrowhead	100 µm	[91]
2	Metal NPs	5, 10, and 20 nm	Au	2–3 V	1 MHz	Au	Nanogap	20 nm gap electrode	[100]
3	Metal NPs	20–30 nm	Au	10 V_pp_	1 MHz	Ti/Au	Interdigitated	1 µm/-/1 µm/5 nm, 200 nm	[71]
4	Metal NPs	60 nm	Ag	10 v	20 MHz	Cr/Au	Gap electrode	5 µm/-/-/50 nm, 150 nm	[97]
5	Metal NPs	50 nm	Ag	1–20 V_pp_	1 Hz–1 MHz	Cr/Au	Quadrapole	-/-/-/5 nm, 100 nm	[4]
6	Metal NPs	40 nm	Au	10 V_pp_	1 MHz	Au	Nanopore	20 µm gap/70 nm Dia/5 nm Au coating	[94]
7	Metal NPs	15–100 nm	Ag/Au	1–20 V_pp_	1 MHz	Au	Nanoelectrode array	80 nm/-/-/50 nm	[74]
8	Metal NPs	40 nm	Ag	7–8 V	1–10 MHz	Au	Si Tips/Au	-	[85]
9	Metal NPs	40 nm	Ag	16 V_pp_	2.5 MHz	Au	-	-/-/-/100 nm	[101]
10	Metal NPs	2, 10, and 100 nm	Au	20 V_pp_	5 MHz	SiC/SWCNTS	Nanotip	540 ± 140 nm	[98]
11	Metal NPs	35, 120 nm	Au	0.6–6 V_rms_	600 kHz	Cr/Ag	Nanoprobe	(150–500 nm in diameter, 2–150 µm in length)	[56]
12	Metal NPs	150 nm	Au	1.9–15 V_rms_	100 kHz	Au	Gap electrode	10 µm/	[93]
13	Metal NPs	60 nm	Au	0.7–2.5 V	1 kHz–1 MHz	Au	Rounded/Rectangular	40–100 nm, 1 µm, 10 µm/-/-/100 nm	[102]
14	Metal NPs	100 nm–200 µm	Silver coated Silica	100–600 V	200 kHz	Al	Needle-shaped electrode	-	[5]
15	Metal NPs	20 nm	Au	3 V	10 kHz–1 MHz	Cr/Pd	Triangular planar electrode	3 µm/-/-/10 nm, 70 nm	[10]
16	Metal NPs	40 nm	DNA coated Au	2.5–2.8 V	4 MHz	Ti/Au	Nanogap electrode	13 nm/-/-/-	[103]
17	Metal NPs	10 nm	Au	9–13 V	1 kHz–1 MHz	Cr/Au	Triangular planar electrode	10 µm/-/-/20 nm, 120 nm	[104]
18	Metal NPs	80, 100, 150 nm	Au	<10 V_pp_	1–5 MHz	ITO	Thin-film electrode	20 µm/-/-/-	[99]
19	Metal NPs	20 nm	Au	3 V_pp_	1 MHz	Cr/Au	Nanogap electrode	200 nm/-/-/5 nm, 30 nm	[95]
20	Metal + nonmetal NPs	5, 10, 22 nm	Au, Cu, W, Al, Si, PSL	12–20 V_pp_	30–70 kHz	Cr/Au	Parallel electrode	-/-/-/2 nm, 120 nm	[73]
21	Metal NP + Ligands	10 nm	Au	4 V_pp_	1 MHz	Ti/Pd	Nanogap electrode	50 nm/-/100 nm/10, 40 nm	[105]
22	Metal NPs	15 nm	Pt	5 V	500 kHz	Au	Microgap electrode	-/-/-/4 µm	[92]
23	Metal NPs	2–4 nm	Pd	1–4 V_pp_	500 kHz–2 MHz	Ti/Au	Coplanar electrode	4 µm/-/-/10, 200 nm	[76]

DEP has emerged as a vital technique for controlling metallic NPs, driving advancements in the creation of nanostructures, as well as in sensing and electronic applications. Its precision in manipulating nanoparticle behavior has propelled both foundational research and real-world innovations across multiple disciplines. With ongoing efforts to refine DEP methods, the possibilities for utilizing metallic NPs in nanotechnology and similar areas are vast.

### 3.2. Non-Conducting Nanostructures

Non-conducting particles, as natural insulators, undergo dielectric polarization in an electric field. Due to their well-defined, uniform, and inert dielectric properties, they serve as model systems for developing, validating, and optimizing novel DEP architectures [106]. Their consistent dielectric behavior and commercial availability make them ideal candidates for the calibration and prototyping of sensors designed for biological applications [107]. Consequently, researchers have utilized DEP as a tool for manipulating non-conducting particles to overcome the limitations of the existing techniques, separation and sorting studies, quantitative force measurements, as well as simulation and modeling studies. A summary of the relevant research and its key details is presented in Table 3.

A novel experimental approach was developed to measure the DEP potential spectrum of colloidal NPs while minimizing the influence of particle shape and size. This method employed confocal laser scanning microscopy to quantify the time-averaged number-density distribution of particles under a DEP force field, enabling the determination of the frequency-dependent dipole coefficient and the crossover frequency for NPs ranging from 63 to 410 nm. By leveraging a statistical mechanics-based framework, this approach enhanced the analysis of DEP response functions and is particularly effective at frequencies below the crossover frequency, where existing methods have limitations to detect small DEP forces [108].

The ultra-high yield of single NPs was achieved by combining pDEP and nDEP forces to enable reversible attachment and detachment of NPs. A 100 nm Au NW electrode was used to manipulate 100 nm red fluorescent beads and 25 nm polyethylene-glycol-coated CdSe/Zn QDs [83]. Additionally, the combination of pDEP and nDEP (10 V_pp_ signal), with the positioning capabilities of an atomic force microscope (AFM), facilitated the precise patterning of 10–30 nm alumina particle arrays on a hexamethyldisilazane-coated indium tin oxide (ITO) glass substrate [109]. AFM-DEP was also utilized in assembling 200 nm polystyrene particles into lines, ellipsoids, and dots [110]. Furthermore, a coaxial AFM probe-DEP tweezer has found its application in the trapping and collection of 20 nm polystyrene and deoxyribonucleic acid (DNA) molecules in a highly conductive (160 mS/m) buffer and an nDEP force in low conductive buffers (6 mS/m) [111].

**Figure 3:**
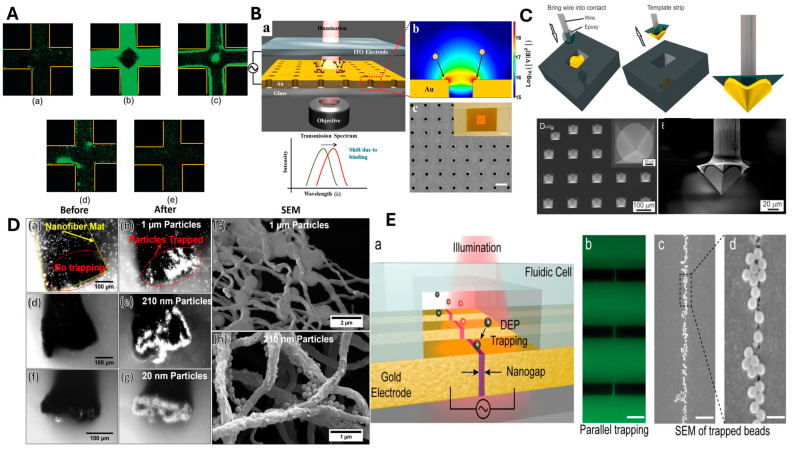
(**A**) Fluorescence images of polystyrene (PS) particles (**a**) without AC fields. With 10 VPP and an AC frequency of (**b**) 1 MHz, (**c**) 5 MHz, (**d**) 7 MHz, and (**e**) 10 MHz. Adapted with permission from [72]. Copyright 2010 American Chemical Society. (**B**) (**a**) Illustration of the experiment for DEP concentration of analyte molecules. (**b**) Simulation of the electric field intensity gradient. (**c**) SEM of the nanohole array [112]. (**C**) Fabrication of gold pyramid, its connection to tungsten wire and SEM images of gold pyramids in the mold and a single pyramid [113]. (**D**) DEP trapping by conductive nanofiber mat: 1 µm PS particles (**a**) without AC fields, (**b**) with AC fields, and (**c**) SEM image; 210 nm PS particles (**d**) without AC fields, (**e**) with AC fields, and (**h**) SEM image; 20 nm PS particles (**f**) without AC fields, and (**g**) with AC fields. Adapted from [86] with permission from The Royal Society of Chemistry. (**E**) DEP trapping of 30 nm PS beads with very-low AC fields. (**a**) Illustration of nanogap electrodes array for DEP trapping. (**b**) Fluorescence image of PS beads trapped across three 20 μm nanogaps. (**c**,**d**) SEM images of DEP trapping of PS beads along the nanogap. Adapted and modified from [114]. Figure 3B,C,E are an unofficial adaptation of articles that appeared in an ACS publication. ACS has not endorsed the content of this adaptation or the context of its use.

Ultra-fast fluorescence correlation spectroscopy was demonstrated as an accurate and rapid characterization technique for colloidal NPs (<50 nm), comparable to the double-layer length scale under varying AC field frequencies (5 kHz to 20 MHz) and a potential of 10 V_pp_. Results revealed two crossover frequencies that strongly depended on particle size and medium conductivity. The fluorescence images of the same are shown in Figure 3A [72].

Ionic concentration-polarization (CP)-based biomolecule preconcentration was combined with DEP dynamic trapping for assessing the binding signal. This addressed the limitation of conventional CP-based biomolecule preconcentration methods which struggle to control the spatial overlap between the preconcentrated plug of biomolecules and surface immobilized antibodies. Biotin-conjugated polystyrene particles (0.8 µm) and fluorescein-tagged avidin D were used with ac AC field of 10 V_pp_ and 100 kHz applied across a DEP electrode array of 25 μm wide and 25 μm gap [115].

A single electrodeless polydimethylsiloxane device was described for both the mixing and de-mixing of 20 nm and 100 nm polystyrene particles, with driving forces generated by 500 V AC, 50 V DC voltages, and electrokinetic driving forces along the channel [116]. A similar approach, incorporating nano-constrictions, was employed for rapid nanoparticle and protein detection [117]. Moreover, programmable manipulation of polymethyl methacrylate (PMMA) with a DEP microfluidic device was achieved [78,118]. The approach of using electrodeless devices or the programmable manipulation of NPs allows for dynamic control over the particles as well as offering scalability for handling larger sample volumes and a higher throughput, making DEP usable for large-scale applications.

A DEP micropipette tip with an agarose gel plug was designed to isolate 200 nm polystyrene particles and high molecular weight DNA (hmw-DNA) to the pDEP high-field region, while 10 μm polystyrene microbeads were directed towards the nDEP low-field region with an AC field of 160 V_pp_ and 10 kHz [84]. The same group, along with researchers from University of California, focused on a more cost-effective alternative for microelectrode patterning for DEP trapping of polystyrene NPs and λ-DNA [119]. They also utilized DEP to rapidly isolate hmw-DNA and NPs to the pDEP high-field region and blood cells to the nDEP low-field region from 20 μL whole blood samples, without the need for preparation [120]. These studies contributed to making DEP-based separation techniques more efficient, cost-effective, and accessible, opening doors for broader applications in medical diagnostics.

An integrated microfluidic Raman system with curved electrodes was used to determine the suspended particle concentration of Tungsten trioxide and polystyrene NPs via DEP, creating both high and low particle concentrations [121]. The issue of a short trapping range in conventional plasmonic traps was addressed with DEP-assisted plasmonic trapping. In this method, long-range DEP forces drew 150 nm polystyrene particles closer to the plasmonic trap, increasing trapping efficiency by three times [122]. Molecular detection on a gold nanohole array surface was enhanced by utilizing DEP to overcome diffusion limitations, enabling real-time, label-free detection of bovine serum albumin (BSA) molecules at 1 pM concentration. Reversible trapping was also demonstrated by alternating the signal frequency between pDEP and nDEP. Figure 3B gives an illustration of the experiment, showing an SEM image of the fabricated nanohole array along with the simulations indicating the analyte molecules being attracted towards the edges of the nanohole due to strong electric field intensity gradient along the rim of nanohole [112]. Additionally, the epifluorescent microscope’s lack of sensitivity to low levels of analytes was overcome by combining it with a DEP microelectrode array, enabling the detection of DNA and 40 nm polystyrene particles [123].

DEP was used to immobilize polystyrene NPs on electrode array tips. The combination of SEM and fluorescence intensity distribution facilitated the quantification of immobilized particles and the determination of the electrode-to-particle diameter ratio for single-particle immobilization [124]. To reduce surface roughness and enhance the trapping efficiency in traditional tips, a template-stripped gold pyramid was fabricated using a conductive and dielectric epoxy mix, and the fabrication process along with the SEM images of the gold-pyramid are shown in Figure 3C. This gold pyramid, combined with an ITO electrode, served as a movable DEP trap capable of trapping 2 μm and 190 nm polystyrene particles, as well as single-walled CNTs [113].

A high-throughput DEP-based filtration technique was demonstrated, enabling selective trapping and efficient recovery of microparticles in the mL/min flow range, with scalability up to 50 L/min by increasing the filter cross-section. Compared to conventional microfluidic DEP devices, the technique achieved a five-orders-of-magnitude increase in throughput while maintaining high selectivity. Additionally, the method demonstrated high reusability, achieving 86–92% recovery rates with pH adjustments. These findings validate the feasibility of DEP filtration in macroscopic porous materials, offering a scalable solution for high-throughput particle separation [125].

A scalable and reproducible DEP-based assembly technique was developed for printing nanostructures with precise dimensional control. By applying an alternating current (AC) field and a direct current (DC) offset voltage, NPs were directed into patterned vias, achieving high-uniformity assembly. The combination of 3D-DEP and electrophoresis generated sufficient forces to assemble silica nanorods (20–200 nm diameter, 500 nm–2 μm spacing) and hybrid silica/gold nanorods for plasmonic applications. Compared to conventional approaches, DEP-driven assembly offers a high-precision, bottom-up, and scalable approach for nanoelectronics, photonics, and biosensing. These findings establish DEP as a powerful platform for nanoscale printing, enabling large-area, high-resolution nanomanufacturing [126].

Novel lab-on-a-chip technology integrated droplet microfluidics with DEP to fabricate uniform H1-DNA polyplexes-based nanomedicine. The biological experiments showed that the DEP-treated NPs maintained their gene transfection capability and exhibited significantly higher transfection efficiency (15%) compared to the control group (4%) in HUVEC cells. DEP improved drug delivery efficiency, streamlined the fabrication, mixing, and separation of NPs, and reduced the processing time (from 10 min to 1 min) associated with droplet microfluidics [127].

A recent study investigated methods to generate a high-electric-field gradient to overcome the Brownian transport of NPs and enable bulk electrokinetic particle trapping. A carbon nanofiber mat sandwiched between copper tapes was used as an electrode, with an ITO slide as a planar electrode. A maximum gradient field-squared of 2.82 × 10^17^ V^2^/m^3^ was simulated around the fiber edges enough to trap 20 nm particles. Polystyrene particles of various sizes (20 nm, 210 nm and 1 µm) were successfully trapped with an applied electric potential of 7 V_rms_, and the SEM images in Figure 3D show the trapped particles around the fiber edges as predicted by the simulations [86].

A cost-effective novel approach was adopted to amplify the localized AC electric field by two orders of magnitude, enabling the rapid trapping of 20 nm colloids and bacteria from a diluted blood sample [128]. Sub-10 nm gaps were created between gold electrodes, promoting the rapid, long-range DEP trapping of 30 nm polystyrene, 40 nm diamond, and 8 nm CdSe QDs with a bias voltage as low as 200 mV. By shrinking the separation between gold electrodes to sub-10 nm using high-throughput atomic layer lithography, instead of e-beam lithography, strong trapping forces over a mm-scale trapping zone were created. The illustration of the nanogaps along with the particle trapping are shown as fluorescence images and SEM images in Figure 3E [114].

A novel statistical image quantifying method was developed to determine nanoparticle electrokinetic parameters. It was compared with traditional methods using 200 nm latex nanospheres in low-conductivity media and a planar castellated electrode array for pDEP [129]. Exploiting the innate differences in dielectric properties of NPs, low-density non-magnetic drug delivery NPs (liposome-based, polymer-based, and hollow silica shell-based) with stealth surface coating were recovered from undiluted human plasma [130]. The selective manipulation of 100 nm polystyrene and 20 nm quantum dot NPs in three degrees of freedom was achieved at gold nanostructures fabricated between electrodes, using the floating AC-DEP force with 8 V_pp_ [131]. Furthermore, assembly of the poly-L-lysine core–shell NPs of 220 nm and 400 nm was accomplished with the assistance of DEP [132].

A novel microfluidic chip with zig-zag/face-to-face electrodes for AC and parallel electrodes for DC was devised to establish a continuous upconcentration flow system for sub-100 nm particles. It successfully achieved upconcentration by a factor of 11 for particles as low as 47 nm at 2 µL/h [133]. The capture efficiency of single particle passage through a 1.4 µm micropore was enhanced by controlling the traffic of a polystyrene particle (780 nm) with AC-DEP (2–10 MHz) [134].

**Table 3:** Summary of research works in DEP of non-conducting nanostructures.

Serial No.	Nanoparticle			Electric Field Signal		Electrode Information			Ref.
	NP Type	Size	Material	Voltage	Frequency	Materials	Types	Length Scales (Gap/Length/Width/Height)	
1	NPs	63, 160, 200, and 410 nm	polystyrene	10 V_pp_	30 MHz	Au/Cr	Coplanar parallel electrodes	27 µm/22 mm/-/0.2 µm	[108]
2	NPs/ QDs	100, 25 nm	Polystyrene/PEG coated CdSe/Zn QDs	8 V_pp_	3 MHz–50 MHz	Au	Nanowire electrodes	10 µm/-/100 nm/-	[83]
3	NPs	10–30 nm	Aluminum oxide	10 V_pp_	1 kHz–10 MHz	ITO	Planar electrodes	(4 mm × 4 mm reservoir)/1 cm/1 cm/1 mm	[109]
4	NPs	20 nm, 210 nm, and 1 µm	polystyrene	7 V_rms_	1 kHz–1 MHz	Carbon nanofiber mat/ITO	Electrospun nanofiber electrode	150 µm gap/Mat–80 µm thick and 3 mm wide	[86]
5	NPs	10–50 nm	polystyrene	10 V_pp_	5 kHz–20 MHz	Au	Quadrupole microelectrodes	20 µm gap/-/-/-	[72]
6	NPs/DNA molecules	20, 10 nm	polystyrene	2 V_rms_	100 kHz–50 MHz	Au/Cr	Co-axial probe electrodes	-	[111]
7	NPs	0.8 µm	biotin/avidin-conjugated polystyrene	10 V_pp_	80–100 kHz	Au	Interdigitated electrode	25 μm/-/25 μm gap/-	[115]
8	NPs	20, 100 nm	polystyrene	500 V	600 Hz	-	Electrodeless	-	[116]
9	NPs/ DNA_molecules_	200 nm, 10 µm	polystyrene/genomic hmw-DNA	160 V_pp_/80 V_pp_	10 kHz/3 kHz	Pt/Au	Nanowire electrode/ring type counter electrode	-	[84]
10	NPs	220, 80 nm	Polystyrene/ tungsten trioxide	15 V	250 kHz–20 MHz	Au/Cr	Curved electrodes	20–80 nm/270 nm/30 nm/200 nm	[121]
11	NPs	150 nm	Polystyrene	0.18 V/µm	10 kHz	Au/ITO	Nanopillar electrode	120 nm height/150 nm radius	[122]
12	NPs/ Bioparticles	190 nm	Polystyrene/BSA	10 V_pp_/6 V_pp_	1 kHz–10 MHz	Au/ITO	Nanohole array/planar electrode	Hole diameter—140 nm/periodicity—600 nm	[112]
13	NPs	100 nm to 2 µm	Polystyrene	1.7 to 13.7 V_rms_	15 kHz	W, Si/ITO	Vertical and conical array/planar electrode	Gap—2 µm/W—500 nm dia, Si—50 nm dia./Height—40 nm	[124]
14	NPs/DNA molecules	40 nm	polystyrene/DNA	20 V_pp_	10 kHz	Pt	Microarray electrodes	80 µm dia/Dimensions—2 mm × 2 mm	[120]
15	NPs/ DNA molecules	200 nm	polystyrene/ λ-DNA	12 V_pp_	6 kHz	Au/Ni	Interdigitated circular electrodes	100 µm/-/-/3 to 5 µm	[119]
16	NPs	5, 20, 40, and 80 nm	Si/Au	12 V	1 MHz/10 MHz	Au, PMMA/Au	Planar electrodes	-/-/-/235 nm	[126]
17	Polymer/DNA	116 nm	H1/DNA plasmids	8 V_pp_	20 MHz	-	Interdigitated electrodes	-	[127]
18	Micro/NPs	0.5, 3, and 4.5 µm	Polystyrene/Graphite	150–600 V_rms_	1 kHz–15 kHz	Stainless steel	Parallel planar electrodes	8 mm/18 mm/8 mm/29 mm	[125]
19	NPs	190 nm, 2 µm,	Polystyrene	10 V_pp_	10 kHz–10 MHz	Au/ITO	Pyramid tip/Planar electrode	70 µm/-/-/-	[113]
20	NPs/ DNA molecules	40 nm	polystyrene/DNA	20 V_pp_/14 V_pp_	10 kHz	Pt	Microarray electrodes	80 µm dia, Patch dimensions—200 µm	[123]
21	NPs	300 nm	PMMA	10 V_pp_	200 kHz	Au/ITO	Fish-bone type/Planar electrode	50 µm/-/30 µm/-	[118]
22	NPs	300 nm	PMMA	10 V_pp_	200 kHz	Au/ITO	Planar electrode	30 µm/2000 µm/30 µm/-	[78]
23	NPs	50 nm/40 nm/50 nm	Anti-FITC/polystyrene/Au	50/100/300 V	50/500/260 kHz	Si	Electrodeless	150 nm/-/-/-	[117]
24	NPs/Bacteria	5 µm and 20 nm	Polystyrene/Staphylococcus aureus and Pseudomonas aeruginosa	15 V_pp_	100 kHz–1.2 MHz	Au/Ti	Quadruple electrode array	-/-/-/235 nm	[128]
25	NPs	200 nm	Polystyrene	1 V	1–4 MHz	Pt	Castellated arrays	5 µm/-/5 µm/100 nm	[129]
26	Polymer/nanomedicine particles	100–200 nm	Polymer, Silica, Liposome	18 V_pp_,15 V_pp_, 8 V_pp_, 12 V_pp_	15 kHz	Pt	Circular electrode array	60 µm diameter	[130]
27	NPs/ QDs	100, 20 nm	Polystyrene/QDs	8 V_pp_	1 MHz	Ti/Au	Microelectrode	10 µm/-/-/55 nm	[131]
28	Core–shell NP	220 and 400 nm	poly-L-lysine shell NPs	5 V_pp_	1 kHz–80 MHz	Ti/Au	Quadrupole microelectrodes	25 µm/-/-/100 nm	[132]
29	NPs/Bioparticles	200 nm	Polystyrene/BSA	10 V_pp_	10 kHz	platinum−iridium/ITO	Tip and Planar electrode	Tip: Size—20 nm, height—15 nm, length—225 μm	[110]
30	NPs	47 nm, 1 µm	Polystyrene	20 V_pp_	10 kHz–1 MHz	Ti/Au	Zig-zag/face-to-face	Placement angle—60°. Zig-Zag gap—6 µm. Face-to-face gap—5 µm. Height—110 nm	[133]
31	NPs	780 nm	Polystyrene	1 V	2 MHz–10 MHz	Pt	Crosswise configuration	1 µm/-/-/60 nm	[134]
32	NPs/ QDs/nanodiamond	30, 10, 190 nm	Polystyrene/Nanodiamond	300 mV/750 mV/400 mV to 600 mV	1 MHz to 10 MHz/1 MHz/100 kHz	Au	Nanogap electrode	1–10 nm/0.8 mm/20 µm/150 nm	[114]

DEP has emerged as a powerful tool for manipulating non-conducting particles, enabling researchers to develop innovative techniques and improve existing technologies. The integration of DEP with traditional approaches has led to breakthroughs, overcoming previous limitations and yielding unexpected results. Additionally, novel statistical methods for analyzing electrokinetic parameters have enhanced image quantification and data analysis. The unique properties of non-conducting particles make DEP invaluable for applications such as calibrating and validating biological sensors, separation and sorting, quantitative force measurements, and simulation studies. As research continues to advance, DEP will likely play an even greater role in shaping the future of electrokinetic manipulation of non-conducting particles.

### 3.3. Nanowires/Nanotubes/Nanorings and Similar

The ability to control the alignment and assembly of one-dimensional nanostructures such as NWs, nanotubes (NTs), and nanorings (NRs) is central to their integration into functional devices. DEP’s versatility in controlling the positioning, alignment, and functionalization of such nanomaterials has made it indispensable for applications across disciplines. Researchers have used them in fabricating flexible electronics including display devices, transistors, memories and logic gates, solar cells, sensors, and nanogenerators [135]; in energy conversion devices [136]; multicolor nanophotonics [137], etc. Here, we review key studies on the assembly, patterning, and sensor integration of NWs, NTs, and NRs through DEP, highlighting recent advancements and the diversity of approaches within this domain. A summary of relevant research is shown in Table 4.

Before exploring the direct applications of DEP in the assembly and patterning of NWs and NTs, exploring several key factors, including frequency, electrode design, and medium properties, is essential. Several studies have highlighted the intricate relationship between the applied frequency, material properties, and assembly behavior, offering valuable insights into the conditions required for controlled nanostructure manipulation.

**Figure 4:**
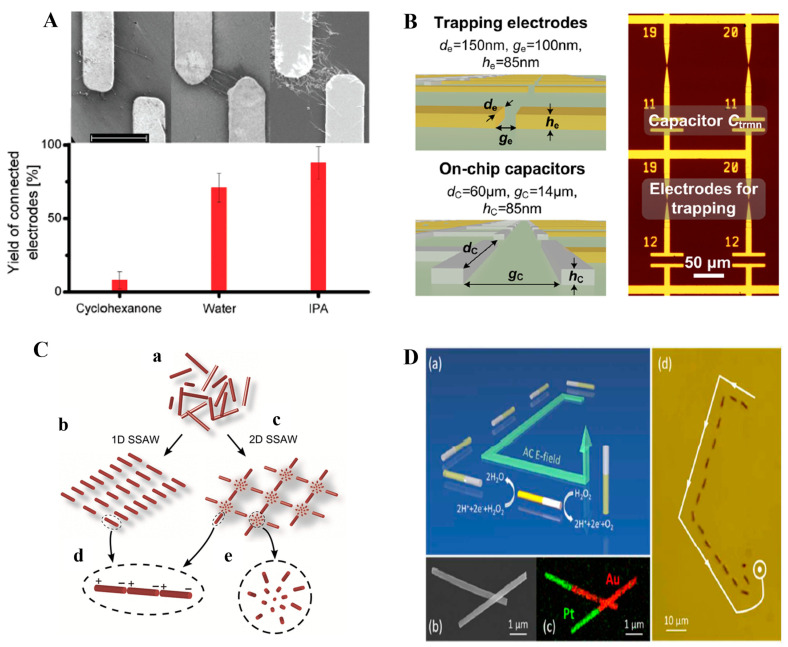
(**A**) Mean electrode connection yield (with 1 SD as error bar) after DEP of CNTs dispersed in cyclohexanone, water, and IPA solution: applied voltage was 2 *V*_TOT_ with 2 µm of oxide layer. Adapted with permission from [142]. Copyright 2010 American Chemical Society. (**B**) Design of a self-limiting dielectrophoretic device featuring a microscopic image of an array with four units, each consisting of a pair of trapping electrodes connected in series with a capacitor. Adapted and modified with permission from [143]. Copyright 2022 American Chemical Society. (**C**) Schematic of patterning technique by SSAW (**a**) randomly dispersed NWs, (**b**) 1D-, (**c**) 2D-SSAW fields formed NW arrays, (**d**) assembled into bundles due to E. field, and (**e**) observed 3D-sparked at the nodes. Adapted and modified with permission from [144]. Copyright 2013 American Chemical Society. (**D**) (**a**) Schematic diagram of Pt-Au catalytic nanomotors manipulations with AC electric fields, (**b**) SEM image, (**c**) energy-dispersive X-ray spectroscopy images of catalytic nanomotors, and (**d**) snapshots of a nanomotor manipulation. Adapted with permission from [145]. Copyright 2018 American Chemical Society.

For instance, Kataoka et al. demonstrated that tuning the frequency (10 kHz to 1 MHz) significantly impacts the rate and quality of Ag NW assembly, optimizing their alignment for specific applications [138]. Similarly, a study using a 3DEP chip showed that higher frequencies (above 1 MHz) improved the conductivity of aligned NWs, suggesting frequency control enhances both assembly and electrical performance [139]. Tao et al. found that the alignment of ZnO NWs and carbon NTs is governed by the combined effects of frequency and electrode gap size, which are crucial for integrating nanostructures into functional devices [140]. Additionally, Abdulhameed et al. explored how medium properties, such as permittivity and conductivity, influence CNT motion during DEP, offering insights for optimizing the process in different environments [141]. Duchamp et al. found that the dielectric permittivity of the solvation shell of CNTs and the pattern of the electric field, determined by the substrate resistivity, lead to differences in the results of DEP. Solvents with a low solvation shell dielectric constant (water and isopropyl alcohol) should be used to separate conducting CNTs, as shown in Figure 4A [142]. These studies underline the importance of precise DEP control for effective NW and NT assembly in device applications.

DEP has enabled the precise assembly and positioning of NWs and NTs for a variety of applications. Studies have demonstrated that by adjusting the frequency and voltage, Ag NWs can be directed between electrode pads or along their edges, which is useful for NW-based sensors and interconnectors [146]. Additionally, a self-limiting DEP process has been developed to achieve 98.5% precision in single-NW assembly, essential for large-scale production of NW-based devices [147]. Cao et al. further advanced the technique by using fringing electric fields to align high-density CNT arrays with a consistent pitch of 50 NTs per micrometer, enabling their application in high-performance nanodevices like field-effect transistors [11]. Furthermore, combining DEP with capillary-assisted assembly allows for precise alignment and secure positioning of NWs, enhancing scalability for flexible electronics [148].

Building on these advancements, Wang et al. developed a reusable electrode method for assembling Ag NWs on flexible PET substrates using sinusoidal AC voltages, achieving ordered arrays and rectangular mesh-like networks through a two-step field rotation, demonstrating potential for low-cost integration in flexible electronics [149]. Similarly, a study on NW-based oscillators employed DEP and magnetic interactions to precisely anchor multisegmented NWs on patterned nanomagnets, enabling synchronized motion for thousands of cycles, with applications in nano-resonators and biochemical sensing [150]. Additionally, a scalable DEP technique for site-selective nanoparticle trapping incorporated capacitors to limit multiple particle trapping, achieving a 70% single-particle yield and offering a precise, scalable solution for nanostructure assembly in large-scale device fabrication [143]. As shown in Figure 4B, the design incorporated a capacitor in series with the electrodes. The impedance between the electrodes is reduced when an NW bridges the gap and the capacitor takes the majority of applied potential, thereby reducing the voltage across the gap and inhibiting multiple particles from being trapped.

The optimization of CNT positioning has also been explored, with research showing that solvent polarity and substrate characteristics play a crucial role in their alignment during DEP, which could improve CNT-based sensors and electronics [151]. Additionally, a study on the charge transport behavior of single CuO NWs revealed their potential for use in sensors and optoelectronics, where understanding the charge transport is critical to device performance [152]. A universal set of parameters for aligning NWs, applicable to various materials, is determined, focusing on factors such as peak-to-peak potential, frequency, the thickness of the silicon oxide layer, grounding of the silicon substrate, and solvent properties for templated cathodic electrodeposition [153]. These studies highlight the versatility of DEP in the assembly and functional integration of NWs and NTs, paving the way for their use in advanced electronic and sensor technologies.

Patterning NWs, NTs, and NRs is crucial for integrating nanostructures into functional devices, and DEP offers an efficient, scalable solution. One method utilizes surface acoustic waves to tune the frequency and assemble NWs into specific geometric patterns, providing a contactless, template-free approach suitable for large-area patterning [144]. A schematic representation of formed patterns is shown in Figure 4C. Another study used DEP to position gold NWs around patterned cylindrical posts, with frequency adjustments allowing for the selective placement of NWs between or around the posts. This reconfigurable technique enables the creation of complex patterns for optical and nanoscale applications [154]. Additionally, a DEP system with dot-matrix electrodes was developed for precise NW alignment, improving capture rates and positioning, making it effective for integrating NWs into electronic devices with varying lengths [155]. Different assembly patterns and controls of the pattern can be achieved by varying the electric field voltage and frequency. These studies highlight the versatility of DEP in creating well-defined patterns essential for advancing nanodevice technologies.

NWs, NTs, and NRs are ideal for sensor applications due to their high surface-to-volume ratio and tunable electrical properties, with DEP enabling their precise assembly into functional sensors. One study integrated CNT sensors into CMOS microsystems, creating an array of individually addressable CNT-based sensors with high sensitivity to pH changes, showcasing DEP’s potential for environmental monitoring and biotechnology applications [156]. This excellent review focused on gas and photosensors, where DEP was used to assemble semiconducting NWs and CNTs with exceptional sensitivity and reproducibility, highlighting DEP’s versatility in sensor fabrication [157].

In addition to enabling assembly and patterning, DEP also allows for the real-time manipulation and in situ characterization of individual nanostructures, offering valuable insights into their behavior and performance. One study demonstrated the manipulation of SnO₂ nanobelts in a microfluidic environment using AC DEP, where pDEP and nDEP forces were used to control the movement of the nanobelts, suggesting new applications in sensors and optoelectronics [158]. Similarly, real-time manipulation of Si NWs was achieved with DEP, with I-V measurements and photoconductivity analysis providing insights into their potential integration into electronic and photonic devices [159]. The movement of catalytic nanomotors can be controlled using a combination of DC field to modulate the speed through electrophoretic and electroosmotic forces while the AC field guides their alignment through an induced dipole, as shown in Figure 4D, which has the potential of powering functional nanomechanical devices [145]. Additionally, the DEP growth of platinum NWs was studied, focusing on the effects of concentration and temperature on their growth, further enhancing the understanding of how DEP can be leveraged for NW-based device fabrication [77]. Together, these studies highlight the crucial role of DEP not only in assembling nanostructures but also in enabling their precise manipulation and characterization for advanced applications.

DEP continues to demonstrate its pivotal role in advancing the fabrication, assembly, and manipulation of one-dimensional nanostructures such as NWs, NTs, and NRs. Through its diverse applications—from patterned assembly for device integration to the development of advanced sensors—DEP offers a scalable, versatile solution for the fabrication of next-generation nanomaterial-based devices. As techniques like surface acoustic wave-assisted patterning, fringing-field assembly, and self-limiting deposition continue to evolve, the potential for DEP in nanomanufacturing will only expand, enabling breakthroughs in electronics, biotechnology, and nanorobotics.

**Table 4:** Summary of research works in DEP of nanotubes/nanorings/nanowires and similar.

Serial No.	Nanoparticle			Electric Field Signal		Electrode Information			Ref.
	NP Type	Size (Diameter/Length)	Material	Voltage	Frequency	Materials	Types	Length Scales (Gap/Length/Width/Height)	
1	Metal NPs/NWs	100 nm (NPs), 60 nm (NWs)	Ag	10–30 V_pp_	10 kHz–1 MHz	ITO	Interdigitated, pillars, and pits	-	[138]
2	NWs/NTs	200 nm/3 µm	ZnO, CNT	5 V	1 MHz	Ti/Au	Narrow gap	1 µm, 4 µm, 5 µm/-/-/5 nm, 30 nm	[140]
3	sc-SWCNTs	-	CNT	10 V	500 kHz	ITO	Rectangular gap electrode	10 µm/995 µm/-/0.14 µm	[141]
4	MWCNTs/NWs	5 nm/200 nm–4 µm	ZnO/TiO_2_/VOX/CNT	2 V	1 MHz	Au	Microelectrode pair	2 µm/-/-/50 nm	[142]
5	NWs	220 nm/30 µm	CuO	6 V	20 kHz	Au	Gap electrode	2.25 µm/-/-/-	[152]
6	NWs	60 nm/40 µm	Ag	-	19–37 MHz	Cr/Au	Gap electrode	75 µm/-/75 µm/50 Å, 500 Å)	[144]
7	NWs	300 nm/2.5 µm	SiO_2_ coated Ag	2–6 V	750 kHz	Ti/Au	Cylindrical post	-/-/-/15 nm, 30 nm	[154]
8	NWs	100 nm/200 µm	Ag	0.6–5 V	10 kHz–10 MHz	Ag	Dot-matrix electrode	50 µm diameter/150 µm gap	[155]
9	NWs	75 nm/22 µm	Si	10 V_pp_	1 kHz–20 MHz	Au	3D-well electrode	150 µm/-/-/70 Cu, 150 Polyamide	[139]
10	NWs	2 nm/-	Au	2–10 V_pp_	2–20 MHz	Cr/Au	Parallel probe electrode	3 µm/5 nm, 50 nm	[146]
11	NWs	30 nm/20, 60 µm	Ag	5–70 V_pp_	1 MHz		Interdigitated electrode	36, 65 µm/-/36, 184 µm/-	[149]
12	SWCNT	-/600 nm	CNT	5 V_pp_	400 kHz	Ti/Pd/Au	Surface microelectrodes	100 nm/-/100 nm/0.2, 15, 15 nm	[11]
13	NWs	50 nm/4–5 µm	Si/InAs/ZnO	3.2 V_pp_	50 kHz	Cr	Interdigitated electrode	2, 4, and 5 µm/-/-/100 nm	[148]
14	NWs	30 nm/1–20 µm	Si	5–20 V_pp_	5 kHz–5 MHz	Ti/Au	Parallel electrode-bars/Interdigitated electrode	10, 20 µm/-/-/2 nm, 50 nm	[151]
15	NWs	240 nm/18 µm	Si	7 V_rms_	500 Hz	Al	Isolated and sparse electrode	-/12 µm/2 µm/50 nm	[147]
16	NWs	151 nm/5.8 µm	Au	2–8 V_pp_	10–50 MHz	Au/Ni/Au	Quadrupole microelectrode	-/-/-/6 nm, 100 nm, 100 nm	[150]
17	NWs	-	Au/MWCNT	4 V_pp_	10 kHz–1 MHz	Cr/Au	Array of electrodes	100 nm/-/150 nm/85 nm	[143]
18	NWs	130 nm/-	Si	0.3–5 V	1 MHz	Ti	Tapered electrode	4 µm/-/-/-	[159]
19	Nanobelt	1–2 µm length/size	SnO_2_	70 V_pp_	5 Hz–10 MHz	Au	Castellated microelectrodes	20 µm/-/-/250 nm	[158]
20	Nanomotor	250 nm/5 µm	Pt–Au	0–50 V_pp_	10 kHz–10 MHz	Au	Quadruple microelectrode	-/-/-/500 µm	[145]
21	NWs/NTs	50–150 nm/4–6 µm	Ni, ZnO, Au, Ag, Sn, Fe_2_O_3_	0.6–6 V_pp_	50–200 kHz	Au	Parallel plate electrodes	-/-/-/400 nm	[153]
22	NWs	23 nm/-	Pt	4 V	100 kHz	Pt/Au	Interdigitated electrode	2–4 µm/-/-/3, 17 nm	[77]
23	NTs	1–3 nm/	CNT	5–20 V_pp_	300 kHz	W-Ti/Pt	Interdigitated electrode	-/-/-/120 nm	[156]

### 3.4. Other Semiconductive Nanostructures

Other semiconductive nanomaterials like graphene, carbon black, quantum dots, indium gallium nitride, etc., have emerging applications due to their unique electronic, optical, thermal, and chemical characteristics. Researchers have used them to create novel biosensors [160], sensors for environmental contaminants monitoring [161], and in high performance photodetectors [162]. In particular, graphene-based semiconductive nanomaterials have great potential in optical and optoelectronic applications, including light-emitting devices, photovoltaics, saturable absorbers for ultra-fast lasers, transparent conductive electrodes, photodetectors, and phototransistors, as well as new and developing photocatalytic applications and biological processes [163]. As a result, researchers utilized DEP to assemble, manipulate, and precisely align such nanostructures to facilitate these applications. A summary of relevant research with key parameters has been listed in Table 5.

**Figure 5:**
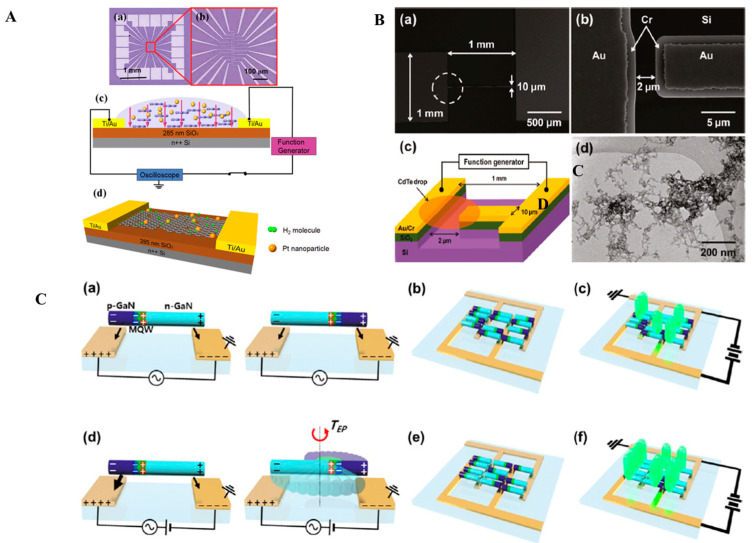
(**A**) (**a**) Optical, (**b**) higher magnification optical microscopy image of the patterned electrode, (**c**) experimental setup schematic, and (**d**) schematic showing assembled GO nanostructures and Pt NPs. Adapted with permission from [164]. Copyright 2015 American Chemical Society. (**B**) (**a**,**b**) SEM image of DEP electrodes: zoomed in view of the electrode gap (**b**) marked by the dotted white circle in (**a**), (**c**) schematic of the experimental setup used for DEP, (**d**) TEM image of the 50 μL CdTe NPs dispersion after DEP. Adapted with permission from [166]. Copyright 2011 American Chemical Society. (**C**) Orientation of nanorod LEDs schematic when aligned: (**a**) cross-sectional and (**b**) top-view images of nanorod alignment using an AC electric field, and (**c**) randomly aligned and with DC voltage approximately half of them turned on; (**d**) cross-sectional and (**e**) top-view images of nanorod LED device using AC electric field with DC offset. Most of them were forwardly aligned due to the intrinsic dipole torque and (**f**) the aligned LEDs were mostly turned on when DC voltage was applied. Adapted with permission from [167]. Copyright 2017 American Chemical Society.

DEP is an effective technique for assembling and manipulating nanostructures with properties suited to advanced sensing, particularly hydrogen detection. Through precise control of voltage, frequency, and processing time, DEP enables the assembly of Pt-decorated graphene oxide (GO) nanostructures between microgap electrodes, achieving around 10% sensitivity at 200 ppm hydrogen at room temperature, making it ideal for applications like gas storage and leak detection [164]. The optical microscope image of the fabricated microgap electrodes and the schematic of the experimental system are demonstrated in Figure 5A. Similarly, a high-performance hydrogen sensor using reduced graphene oxide decorated with Pt–Pd NPs has been developed, exhibiting stable and repeatable responses due to mechanisms like carrier donation and lattice expansion [165]. Enhanced by factors like nitrogen as a carrier gas, DEP-assembled sensors demonstrate adaptable and efficient hydrogen detection across varied environments, highlighting DEP’s versatility in creating responsive, high-performance sensors for diverse applications.

DEP has also been applied to precisely align SrTiO_3_ NPs between microelectrodes, enabling electrical measurements that reveal conduction mechanisms, such as hopping and tunneling, dependent on temperature [168]. This setup emphasizes DEP’s utility in forming semiconductive nanostructures with specific electronic properties. Similarly, self-assembly capabilities in DEP have facilitated the formation of photoconductive microbridges using CdTe NPs [166]. The DEP electrode along with the experimental setup and TEM images of NPs dispersion after DEP where particles formed chain are shown in Figure 5B. These size-quantized particles self-organize into chains, amplifying polarization volume and allowing low-voltage assembly while retaining optical properties. The organized NP chain structure opens avenues for MEMS, optoelectronic devices, and sensors.

DEP’s flexibility extends to assembling large-scale devices, as seen with green InGaN nanorod LEDs [169]. Combining techniques such as nanosphere lithography and DEP, millions of nanorod LEDs are arranged between interdigitated electrodes over large areas, achieving scalable, polarized lighting. The devices, though under development, show potential for high-luminance applications like displays. An advanced DEP method using DC-offset AC and pulsed-DC electric fields further enhances this potential by achieving 1.8-times greater electroluminescence intensity than conventional AC-DEP [167]. The schematic of the aligned nanorod orientation is shown in Figure 5C. The pulsed-DC field significantly improves nanorod alignment, leading to increased brightness and current under DC operation, which is promising for a high-efficiency, scalable surface and formable lighting. DEP has also proven effective in fabricating ZnO NP-based ultraviolet sensor arrays with low operating voltage and robust environmental stability [170]. This capability highlights DEP’s potential for developing sensitive, low-power photodetectors across various applications.

Innovative uses of DEP also include growing carbon black wires in acrylate monomers, where voltage, frequency, and temperature impact wire growth and shape, transitioning from linear to fractal structures at higher frequencies [171]. This approach holds promise for creating conductive adhesives suitable for semiconductor packaging. Additionally, carboxylic-functionalized CNTs have been suspended and arranged into controlled gap networks between parallel electrodes, aided by NT interactions in DEP setups [172]. The successful alignment of CNTs showcases DEP’s potential in forming conductive nanostructures with custom geometries for electrical applications.

Beyond assembly, DEP has enabled the precise trapping and manipulation of onion-like carbon (OLC), or carbon nano-onions, which show promise for sensors and nanoelectronics [173]. By optimizing electric field parameters, OLCs are efficiently positioned between microelectrodes, showcasing DEP’s adaptability. Integrating DEP with Raman spectroscopy has further enabled in situ analysis of suspended particles like WO_3_ NPs [121,174]. DEP concentrates particles within specific regions, enhancing Raman analysis of type and concentration, a technique with broad potential for biosensing and particle interaction studies.

DEP’s versatility extends to optofluidic, where it modulates optical properties by controlling nanoparticle distributions near waveguides, impacting light transmission. Electric field tuning positions particles like tungsten trioxide and silica around waveguides, a method paving the way for responsive optofluidic sensors [175]. Another approach combines DEP with ACEO to position QDs on nanowire arrays [176], enhancing biosensor sensitivity by targeting bioanalytes. DEP’s capacity for fine particle control opens opportunities for QD-based biosensors with improved detection.

In NP sorting, DEP has shown effectiveness in separating ZnO particles by shape under low-frequency AC fields [177], achieving distinct placements of rods and cubic forms in an acetone suspension. This precision sorting technique could benefit nanoparticle purification processes, while DEP’s phase-separating ability in graphene oxide dispersions supports microstructure engineering for optical applications [178]. Additionally, DEP’s selectivity based on dielectric properties is utilized in an electrokinetic platform to recover drug-delivery NPs from plasma [130]. This platform preserves plasma protein integrity, enabling the isolation and further analysis of particles. Such a method provides a new avenue for assessing NP stability within biological systems, which is invaluable in drug delivery research.

Through its diverse applications, DEP showcases its transformative potential in creating semiconductive nanomaterial-based sensors and devices with customizable properties, making them ideal for a variety of advanced sensing applications. For more studies and a broader perspective, readers are encouraged to read the following review study which emphasizes the effectiveness of DEP as a bottom-up approach for fabricating nanomaterial-based sensors [157].

**Table 5:** Summary of research works in DEP of other semiconductive nanostructures.

Serial No.	Nanoparticle			Electric Field Signal		Electrode Information			Ref.
	Type	Size (Diameter/Length)	Material	Voltage	Frequency	Materials	Types	Length scales(Gap/Length/Width/Height)	
1	Nanostructure + NPs	1 nm GO/15 nm Pt	Graphene Oxide/Platinum	2, 5, and 10 V_pp_	100, 500, 1000 kHz	Ti/Au	Microgap electrode	4 µm/-/-/-	[164]
2	Nano-onions	5 nm	Carbon	3–20 V_pp_	1, 100, and 1000 kHz	Au	Interdigitated microelectrode	5 µm/6760 µm/5 µm/-	[173]
3	NPs	21.4 nm	Strontium titanate	5 V	1 MHz	Al	Probe electrodes	100 nm/-/-/60 nm	[168]
4	NPs	80 nm& 210 nm	Tungsten trioxide and Polystyrene	15 V	100 kHz–20 MHz	Cr/Au	Curved microelectrode	-/-/-/50, 100 nm	[121]
5	Nps	4.2 nm/-	Cadmium telluride	4–10 V	100 kHz	Cr/Au	Microgap electrode	2 µm/-/-/-	[166]
6	Nanorods	500 nm/2.5 µm	Indium gallium nitride/Gallium nitride	2.8–21 V_rms_	100–950 kHz	Au	Interdigitated microelectrode	2.5 µm/-/3 µm/-	[169]
7	NPs	20–100 nm	Zinc oxide	10 V	300 kHz	Ti/Pd	Gap electrode	800 nm/200 nm/45 nm/5, 40 nm	[170]
8	CBNPs	30 nm/-	Carbon black	12–225 V	10 Hz–10 kHz	Cu	Triangular planar electrode	2–30 mm/-/-/88 µm	[171]
9	MWCNTs	40–60 nm/5–15 µm	Multiwall carbon nanotube	20 V_pp_	5 MHz	Cr/Au	Parallel electrode	1–10 µm/10 mm/5 mm/30, 100 nm	[172]
10	Nanorods	500 nm/3 µm	Indium gallium nitride/Gallium nitride	21 V_rms_	950 kHz	Ti/Au	Interdigitated microelectrode	3 µm/0.7 cm/0.6 cm/20, 200 nm	[167]
11	NPs	300 nm/15 µm	Zinc oxide	40 V_rms_	1 kHz	Au	In-plane electrode	150 µm/-/-/-	[177]
12	NPs	1 nm/5.69 µm	Graphene Oxide	20 V_pp_	10 kHz	ITO	Interlaced electrode	1 mm/-/1 mm/-	[178]
13	NPs	450 nm, 80 nm/-	silica and tungsten trioxide	15 V_pp_	0.1–250 MHz	Cr/Au	Curved microelectrode	20 µm/17 mm/-/-	[175]
14	NPs	60 nm, 109 nm, 10–20 nm/10 nm	Polystyrene/Nanoliposomes/Micelles/DNA strands	2–18 V_pp_	15 kHz	Pt	Microelectrode	218 µm gap/60 µm diameter	[130]
15	NPs	10 nm QD/300 nm, 5 µm Au Nanowires	Cadmium Selenide, Zinc Sulfide QDs/Gold NWs	20 V_pp_	50–700 kHz	-	Parallel microelectrode	20–50 µm/-/-/-	[176]
16	NPs	3–8 nm	Platinum-palladium	10 V_pp_	1 MHz	Ti/Au	Triangular probe electrodes	2 µm/-/-/5 nm, 90 nm	[165]

## 4. Discussion and Conclusions

This review focuses on the DEP phenomena of NPs, providing a theoretical foundation to understand the processes and challenges occurring at the nanoscale, including the need for high field gradients to overcome Brownian motion and the presence of electrohydrodynamics. This article summarizes the recent developments and applications of NP DEP platforms over the past 15 years, a period characterized by exciting challenges and advancements in the manipulation and separation of increasingly smaller objects using DEP. With the current trend in DEP research, as illustrated in Figure 1, this focused review aims to guide researchers towards areas that warrant the most attention for future practical applications. Over the years, the field has made significant progress, pushing the boundaries of unique developments and applications in this area.

DEP is a highly effective technique for manipulating NPs within microfluidic systems. It enables precise sorting, trapping, transportation, and characterization of NPs. Additionally, DEP plays a significant role in the fabrication of electronic and optical devices by utilizing NPs as fundamental components. The technique also allows for the systematic arrangement of particle arrays in specific patterns and positions, enhancing their application in nanotechnology. By incorporating these fundamental developments, DEP has substantial potential in the biomedical field, particularly in handling small biological entities such as DNA, viruses, vesicles, supramolecular structures, molecules, bacteria, and proteins.

By providing precise control at the nanoscale, DEP opens new avenues for research and innovation, addressing scientific challenges that conventional methods struggle to resolve. For example, DEP is increasingly recognized as a powerful and emerging tool in biomedical research, particularly in areas like liquid biopsy and antimicrobial resistance (AMR) diagnostics. In liquid biopsy, DEP can separate critical disease markers, such as circulating tumor cells and extracellular vesicles, from complex biological fluids without requiring labels, making it valuable for early-stage cancer detection and non-invasive patient monitoring [179,180]. In the context of AMR, DEP enables the rapid sorting of live and dead bacterial populations by exploiting their distinct dielectric properties, offering a quicker and more direct alternative to conventional antibiotic susceptibility testing [181,182]. Moreover, though proteins were long considered too small to be influenced by DEP, research by Hölzel and Pethig [183] showed that under specific conditions—namely, when suspended in low-conductivity solutions like deionized water and subjected to high-frequency, non-uniform electric fields in the megahertz range—proteins do exhibit DEP movement. These insights open new possibilities for DEP in protein analysis, expanding its relevance in diagnostics and point-of-care technologies [184]. These technological trends and emerging directions in AC-DEP applications within microfluidics offer readers a clearer vision of where the field is heading.

Controlling NPs using DEP requires precise management of electric field gradients. While DEP technology for various micro-sized particles is well established, its effectiveness with sub-micron and nano-sized particles is still developing and requires significant attention for practical applications. This limitation arises from the cubic nature of the DEP force relative to particle size; consequently, a much stronger force is needed to manipulate smaller particles. A strong electric field, combined with a conductive medium, can lead to temperature increases within the system. This rise in temperature can result in electrolysis and electrode deterioration, which may compromise the system’s efficacy. Therefore, the careful design of electrodes and flow channels is essential to create significant localized field gradients capable of handling nano-sized particles. Such designs would allow for moderate voltages to be applied without a substantial increase in temperature, thereby enhancing the effectiveness of nanoparticle manipulation, separation, trapping, and enrichment. Fortunately, advancements in nanofabrication are facilitating the meticulous design of electrodes and flow channels [69]. However, they use nanofabrication techniques to design high-resolution electrodes, which are sometimes costly and challenging to scale for mass manufacturing [68]. Research is progressing towards overcoming the challenges associated with DEP for NPs.

The efficiency and selectivity of AC-DEP are strongly governed by the frequency of the applied electric field, as different particles exhibit unique dielectric behaviors across frequency ranges [185]. For instance, gold nanoparticles (AuNPs) showed significant changes in assembly morphology when the frequency was varied from 100 Hz to 10 MHz, with higher frequencies producing straighter, more compact nanoribbons [91]. Similarly, in nanowire (NW) assembly, lower frequencies increased the rate of alignment, while frequencies above 1 MHz enhanced conductivity and structural integrity [138,139]. These observations underscore the importance of accurately selecting the operational frequency, particularly around the crossover point where the CM factor transitions from positive to negative—determining whether particles experience attraction or repulsion in non-uniform fields. Frequency tuning must account for particle size, surface conductivity, and medium properties to ensure effective manipulation. Common optimization strategies include sweep-frequency testing, dielectric property modeling, and real-time impedance spectroscopy [186,187,188,189]. However, despite its central role, standardized guidelines for frequency selection are still lacking, presenting a key challenge for reproducible, scalable AC-DEP system design.

To overcome challenges related to scalability, integration, and reproducibility in AC-DEP microfluidic systems, several forward-looking solutions can be considered. One promising approach is the use of modular and scalable device designs, which enable parallel operation of multiple DEP units to increase throughput without compromising control [190]. Integrating reconfigurable electrode arrays and on-chip electronics can enhance system adaptability and allow for precise, real-time manipulation of electric field conditions. For improved reproducibility, there is a need to establish standardized fabrication protocols and calibration procedures, particularly concerning electrode geometry, frequency tuning, and medium conductivity [191,192]. The adoption of low-cost, high-resolution manufacturing methods—such as nanoimprint lithography or additive printing—could also support consistent device production at scale [193]. Additionally, embedding real-time monitoring tools, such as impedance sensors or optical feedback systems, can help track and adjust DEP performance dynamically, reducing variability between experiments [194]. These strategies, collectively, can contribute to making AC-DEP platforms more robust, automated, and ready for real-world deployment across various applications.

Currently, DEP-based nanoparticle systems are hindered by low-throughput, which limits their potential applications [35]. Several examples of “high-throughput” DEP devices from the literature have throughputs no greater than 10 nL/min for particles no smaller than 20 nm [23,116,195]. These volumes are still relatively low, thereby implementations remain at the laboratory scale, and further development is necessary to enable these systems for commercial use. Many applications do not require processing large sample volumes; however, in scenarios that demand continuous separation and identification—such as in some biomedical applications—it is crucial to address the throughput limitations promptly through ongoing improvements. To overcome throughput limitations, it is essential to use specifically engineered designs for DEP systems. Incorporating 3D electrodes is highly desirable as it increases the effective working surface area for DEP manipulation. Moreover, combining traditional techniques such as hydrodynamic, optical, and acoustic methods can create synergistic effects on throughput that improve the manipulation of NPs and enhance the forces involved in trapping or separating them. This review summarizes how multiple physical fields could be utilized to produce synergistic effects [196]. For example, researchers have utilized DEP and deterministic lateral displacement, which makes it possible for exosomes to differentiate from big extracellular vesicles (such as ectosomes and apoptotic vesicles) [197].

## Figures and Tables

**Table 1:** The estimated minimum gradient of the field-squared needed for DEP to overcome the Brownian motion of particles.

Particle Size (nm)	Gradient of Field-Squared (V^2^/m^3^)
10	1.5 × 10^18^
100	4.8 × 10^15^
1000	1.5 × 10^13^
